# Genetic Variation in Jamaican Populations of the Coffee Berry Borer, *Hypothenemus hampei*

**DOI:** 10.1093/gbe/evae217

**Published:** 2024-11-01

**Authors:** Mohammed Errbii, Ameka Myrie, Dwight Robinson, Eva Schultner, Lukas Schrader, Jan Oettler

**Affiliations:** Institute for Evolution and Biodiversity, University Münster, Münster 48149, Germany; Zoologie/Evolutionsbiologie, Universität Regensburg, Regensburg 93053, Germany; Department of Life Sciences, The University of the West Indies, Mona, Kingston, Jamaica; Zoologie/Evolutionsbiologie, Universität Regensburg, Regensburg 93053, Germany; Institute for Evolution and Biodiversity, University Münster, Münster 48149, Germany; Zoologie/Evolutionsbiologie, Universität Regensburg, Regensburg 93053, Germany

**Keywords:** genome evolution, transposable elements, population bottleneck, pest insect, bark beetles

## Abstract

The coffee berry borer (CBB) *Hypothenemus hampei* was first described in Africa in 1867 and has spread to all major coffee-producing regions worldwide, including Jamaica. Using long-read sequencing, we produced a new high-quality reference genome (172.7 Mb) for the Jamaican strain of the CBB, with 93% of the genome assembled into 14 scaffolds. Whole genome sequencing of pooled samples from different populations across Jamaica showed that the CBB harbors low levels of genetic diversity alongside an excess of low-frequency alleles, indicative of a recent genetic bottleneck. The analyses also showed a recent surge in the activity of transposable elements (TEs), particularly LINE/R1 and LTR/Gypsy elements, within CBB populations. Our findings offer first insights into the evolutionary genomics of CBB populations in Jamaica, highlighting the potential role of TEs in shaping the genome of this important pest species.

**Significance**

The coffee berry borer (*Hypothenemus hampei*) poses a significant threat to coffee farms globally. Here, we provide a high-quality reference genome for the Jamaican strain of *H. hampei* and conduct the first population genomic analyses of the beetle. Our findings indicate low genetic diversity in Jamaican CBB populations, likely due to founder effects, alongside an increased activity of transposable elements. Our study paves the way for future investigations aimed at understanding spatiotemporal variations in genome dynamics.

## Introduction

Human activity has resulted in the worldwide spread of many agri- and aquacultural pest species as well as vectors of diseases, with major consequences for ecology and economy (Jarju et al. 2009; Chapman et al. 2015; Cole et al. 2019; Johnson and Manoukis 2020). To understand the ecological and economic impacts that unfold following the introduction of species to novel habitats, it is necessary to study how such populations evolve. Genetic bottlenecks are expected to limit evolvability by substantially reducing effective population size and genetic variation (Frankham et al. 1999; Tsutsui et al. 2000; Puillandre et al. 2008). However, despite these constraints, introduced populations can adapt to new environments and can successfully cope with biotic and abiotic challenges (Frankham 2005; Schrieber and Lachmuth 2017).

The coffee berry borer (CBB), *Hypothenemus hampei* (Coleoptera: Curculionidae: Scolytinae), is a notorious pest species that thrives where coffee is grown (with the exception of Nepal and Australia) and impacts the economy of millions of people (Moreno-Ramirez et al. 2024). Current pest management strategies are complex, expensive, and often ineffective (Johnson et al. 2020). CBB infestations are typically attributed to anthropogenic factors (Chapman et al. 2015), including migration from abandoned or feral coffee fields (Johnson and Manoukis 2020). Populations of this species are expected to have limited genetic variation (Andreev et al. 1998) due to founder effects and high levels of inbreeding from obligate brother–sister mating (Andreev et al. 1998; Infante et al. 2009). However, this prevalent inbreeding may also benefit the CBB, as sib mating promotes the colonization of nearby coffee berries and facilitates its spread (Gil et al. 2015a). *Hypothenemus hampei* is functionally haplodiploid (Brun et al. 1995), rendering selection more efficient because all expressed alleles are fully exposed in the functionally haploid males, including any with negative fitness effects. This effective purging of recessive deleterious alleles may compensate for potential negative effects of inbreeding in *H. hampei*, much like in other haplodiploid insects (Miller and Sheehan 2023).

Despite exhibiting low levels of genetic variation (Benavides et al. 2005; Gauthier 2010; Gil et al. 2015b), CBB populations can adapt to novel environmental conditions. For example, insecticide resistance has evolved repeatedly in CBBs in New Caledonia (Olivier Brun and Maxwell Suckling 1992; ffrench-Constant et al. 1994) and Jamaica (Witter-Newell 2008), underscoring this species’ ability to overcome control measures. The first draft genome of *H. hampei* provided significant insights into gene families involved in various biological functions, such as detoxification, defense, and insecticide resistance (Vega et al. 2015). Subsequent genomic studies explored additional aspects of the species’ genome biology, focusing on the annotation and analysis of transposable element (TE) dynamics (Hernandez-Hernandez et al. 2017) and the evolution of its chemosensory receptor gene repertoire (Navarro-Escalante et al. 2021). While these efforts greatly enhanced our understanding of *H. hampei*'s biology, they relied on relatively fragmented genome assemblies.

To better understand this major pest, we studied CBB populations introduced to the Jamaican lowlands in 1978 (Reid 1983), around 180 generations ago (assuming ca. 5 generations per year under favorable conditions (Jaramillo et al. 2011; Giraldo-Jaramillo et al. 2018; Hamilton et al. 2019; Johnson et al. 2020). Using a new high-quality genome assembly of the Jamaican CBB strain, we assessed genetic variation and differentiation across four populations, two of which were used previously to estimate CBB activity and infestation rates (Myrie et al. 2023). Furthermore, we explored the introduction history of the CBB into Jamaica and the molecular mechanisms associated with genome dynamics in this species. Our findings reveal low levels of genetic variation and an excess of rare variants, consistent with the recent introduction of the species to Jamaica. Additionally, analysis of TE dynamics indicates recent surges in TE activity within the studied populations. Our study provides high-quality genomic resources and first insights into population genomic dynamics of the most economically significant pest insect of the most profitable hot beverage worldwide (Gauthier 2010; Infante 2018).

## Materials and Methods

### Sample Collection and Sequencing

The beetles used to generate the reference genome for this study were collected in Hopewell, Jamaica (18.03674°N, 76.67991°W), and transferred to the laboratory at the Universität Regensburg, Germany, with permission from the Jamaica Agricultural Commodities Regulatory Authority in Jamaica. In the lab, berries were opened, all stages (eggs, larvae, and pupae) and adult beetles were transferred to individual glass vials already containing 10 mL artificial diet (Vega et al. 2011), and formaldehyde solution min. 37%, added as a microbial inhibitor. The stock cultures were monitored weekly, and when they were too crowded or moist, soft forceps were used to transfer the CBBs (all stages) to a fresh culture. The vials were stored at 27 °C/21 °C under a 12 h/12 h cycle in a dark climate chamber of 100% air circulation and humidity.

High molecular weight genomic DNA was extracted from a pool of 25 adult female beetles using a modified salting-out method (Miller et al. 1988). Briefly, the samples were homogenized in TNES buffer (400 mM NaCl, 20 mM EDTA, 50 mM Tris, pH 8.0, 0.5% SDS) supplemented with Proteinase K and incubated at 55 °C. Nucleic acids were precipitated using ethanol and NaCl, and the resulting pellet was purified through a series of ethanol washes before being resuspended in TE buffer. The sample was then treated with RNase A to remove RNA contaminants. Long-read libraries were generated using Oxford Nanopore Technologies’ (ONT) Ligation Sequencing Kit SQK-LSK110 as described before (Errbii et al. 2024). A total of 9.35 Gb of ONT long-read data (4.69 M reads with a N50 of 6.4 kb) was generated using three FLO-MIN106 flow cells that were sequenced on a Mk1C with fast base-calling.

For genome dynamics analyses, whole genome sequencing data of pools of individuals from four different Jamaican populations were analyzed. Coffee-producing areas in Jamaica are categorized by elevation as follows: Lowlands (<457 meters above sea level [masl]), Highlands (457 to 914 masl), and Blue Mountains (>914 masl). These areas vary in temperature, elevation, rainfall, and humidity. Lowland areas experience high temperatures (25 to 31° C), low humidity, and low rainfall. Highland areas have intermediate conditions with humidity ranging from 60% to 80%, monthly maximum rainfall of 632 mm, and mean temperatures between 18 and 23° C (Myrie et al. 2023). The Blue Mountain areas have the highest humidity (80% to 88%), a maximum monthly rainfall of 339 mm, and the lowest mean temperatures ranging from 16 to 23° C (Myrie et al. 2023).

The CBBs were collected in Jamaica in December 2019 in Kew Park (KP; 18.2593°N, −77.9484°W) in the Lowlands, Baron Hall (BH; 18.2175°N, 77.3767°W) and Mocho (MO; 18.0232°N, −77.3597°W) in the Highlands, and Rosehill (RH; 18.0806°N, −76.73849°W) in the Blue Mountains. Berries with a CBB entry hole were randomly selected and handpicked across the farms. The berries were dissected in the field, and the CBBs were stored in 100% ethanol and then brought to the Universität Regensburg, Germany.

A CTAB method (modified from Sambrook and Russell 2001) was used to extract DNA from pooled samples of adult female beetles (BH [*n* = 40 females], Rose Hill [*n* = 40 females], MO (*n* = 33 females), and KP [*n* = 21 females]). One hundred and fifty base pair paired-end Illumina NovaSeq sequencing of the four pools was performed at the Cologne Center for Genomics to an average coverage of >60× (supplementary table S1, Supplementary Material online).

### Reference Genome Assembly and Annotation

*Fitlong* (v0.2.1) (https://github.com/rrwick/Filtlong) was used to process the raw reads, and a total of 4.8 Gb of long reads (approximately 30× coverage, assuming a genome size of ca. 163 Mb; Vega et al. 2015) were selected using 181 M reads. Contigs were assembled with *flye* (v2.9-b1778) (Lin et al. 2016) with --*nano-hq*, which generated 127 contigs covering 172.36 Mb. Contig N50 was 13.94 Mb, and 90% of the assembly contained the 14 largest contigs (L90 = 14). *dentist* (v3.0.0) (Ludwig et al. 2022) with *join-policy: contigs* was used for further long-read scaffolding and gap filling, which reduced the 127 contigs to 116 scaffolds. We used *NextPolish2* (Hu et al. 2024) for five rounds of long-read polishing, followed by *pilon* (v1.24) (Walker et al. 2014) for ten rounds of polishing using filtered pool-seq short-read data from the BH sample (supplementary table S1, Supplementary Material online). The assembly contained 172,680,286 bp with 13.97 Mb scaffold N50 and an L90 of 13. A comparison of the assembly was made against the endopterygota_odb10 database with *BUSCO* (v5.1.2) (Simão et al. 2015), confirming assembly completeness (98.9%) (C:98.9%[S:98.4%,D:0.5%],F:0.3%,M:0.8%,n:2124). To further assess the completeness of Hham4.1, we screened the assembly for four known Coleopteran telomeric repeats (AACAGACCCG, AACCC, AACCT, and ACCTG) (Brown et al. 2023) using tidk—the Telomere Identification Toolkit (https://github.com/tolkit/telomeric-identifier). The analysis revealed an enrichment of three of these motifs (AACCC, AACCT, and ACCTG) at either one or both ends of the 14 largest scaffolds (supplementary fig. S1, Supplementary Material online).

To remove duplicated scaffolds, *funannotate clean* (https://funannotate.readthedocs.io/en/latest/) was used followed by *funannotate sort* to sort and rename scaffolds; finally, *funannotate mask* was used to mask repeats in the assembly. Protein coding genes were annotated using the 14.4 Gb published RNA-seq data of male and female beetles (SRA accessions: SRR11858905 and SRR11858906) and the *funannotate* pipeline. RNA-seq data were cleaned with *Trimmomatic* (Bolger et al. 2014) and mapped to the assembled genome using *STAR* (v2.7.3.a) (Dobin et al. 2013). Homology-based gene predictions were generated for the CBB with *GeMoMa* (v1.8) (Keilwagen et al. 2019), using gene predictions from *Tribolium castaneum* (GCF_000002335.3), *Sitophilus oryzae* (GCF_002938485.1), *Dendroctonus ponderosae* (GCF_020466585.1), *Coccinella septempunctata* (GCF_907165205.1), and *Harmonia axyridis* (GCF_914767665.1) as reference. Consensus gene predictions, integrating de novo and homology-based predictions and the transcriptomic evidence, were generated with *funannotate update*. Functional annotations were added using *interproscan* (v5.56-89.0) (Jones et al. 2014), and orthogroups were inferred with *eggnog_mapper* (v2) (Cantalapiedra et al. 2021). Finally, *blobtools2* (Laetsch and Blaxter 2017) was used to identify contaminating scaffolds in the assembly. Scaffolds from the assembly that were likely of bacterial origin or that represented the mitochondrial genome were removed, reducing the genome assembly to 114 scaffolds.

The final assembly Hham4.1 for the CBB has 172,680,286 bp in 114 scaffolds. Gene prediction yielded 15,899 genes and 18,624 transcripts, of which 12,225 could be assigned to an *EggNog* orthogroup. Seven thousand eight hundred and eighy-eight transcripts were annotated with Gene Ontology (GO) terms, and 13,811 were annotated with *InterPro* domains. A comparison of the protein annotation against the endopterygota_odb10 database with *BUSCO* (v5.1.2) showed that 97.3% of BUSCOs were present as complete in the protein annotation (C:97.3%[S:96.4%,D:0.9%],F:0.3%,M:2.4%,n:2124).

### Annotation of TEs

To annotate TEs and to produce a TE library, we first generated 1,574 de novo predictions for *H. hampei* using *RepeatModeler2* (Flynn et al. 2020). These de novo repeats were curated using *MChelper* (Orozco-Arias et al. 2023), which automates the TE library curation process, yielding 691 nonredundant curated repeats. Then using the *pfam_scan.pl* script (https://github.com/gpertea/gsrc/blob/master/scripts/pfam_scan.pl) and the Pfam database (v35) (Mistry et al. 2021), we screened the nonredundant library of curated sequences for proper host genes that we discarded. The resulting 687 de novo repeat models were then classified using the *repeatclassifier* module from *RepeatModeler2*. The resulting library of de novo repeats was then combined with arthropod-specific repeats from RepBase (release 27.03) and Dfam (release 3.7), and the Coleoptera-specific repeats (Petersen et al. 2019) to produce a final TE library for *H. hampei*. The final library containing 29,723 sequences was used to annotate repeats in the new genome of *H. hampei* with *RepeatMasker* (v.4.0.7) (https://www.repeatmasker.org) (options: -s -a -gff -inv -excln -no_is -nolow -norna -cutoff 250).

The genomic distribution of TEs and exons was assessed using gene and repeat annotations with the help of *BEDtools* (v2.4.37) (Quinlan and Hall 2010) and *BEDOPS* (v2.4.37) (Neph et al. 2012), and subsequently visualized in R (v4.0.2) (R Core Team 2020).

### Read Mapping

*FastQC* (v0.11.7) (https://www.bioinformatics.babraham.ac.uk/projects/fastqc/) was used to inspect the quality of the raw reads, and *Trimmomatic* (v.0.38) (Bolger et al. 2014) was used to filter out short and low-quality reads (options: ILLUMINACLIP:NexteraPE-PE.fa:2:30:10 SLIDINGWINDOW:4:20 MINLEN:40). The reads were then mapped to the reference genome using *BWA-MEM* (v0.7.17) with default parameters (Li and Durbin 2009), and the quality of the alignments was evaluated with *QualiMap* (v2.2.1) (Okonechnikov et al. 2016). For pool-seq analysis, duplicate reads were removed using *SAMtools* (v1.7) (Li et al. 2009).

### Population Genomic Metric Estimation

Nucleotide diversity (*π*), Watterson's theta (*θ*), and Tajima's *D* were estimated using *PoPoolation*, which accounts for the bias introduced by pooling and sequencing errors (Kofler et al. 2011a). Genetic differentiation (*F*_ST_) was estimated using *PoPoolation2* (v1.201) (Kofler et al. 2011b), following a pipeline described previously (Errbii et al. 2021).

For *π*, Θ, and Tajima's *D*, the alignment bam files were first converted into a mpileup file using *SAMtools*. These mpileup files were filtered for indels using the *identify-genomic-indel-regions.pl* and *filter-pileup-by-gtf.pl* perl scripts under *PoPoolation*. Then, using the *PoPoolation Variance-sliding.pl* script (options: --min-count 2 --max-coverage 112 for BH, 105 for KP, 92 for MO and 117 for RH --pool-size 40 for BH, 21 for KP, 33 for MO and 40 for RH), we estimated *π*, *θ*, and Tajima's *D* in 100-kb nonoverlapping windows.

For *π* at synonymous (*π*_S_) and nonsynonymous (*π*_NS_) sites, we used the mpileup files, and the codon and nonsynonymous codon length tables from *PoPoolation*. We then used the *PoPoolation Syn-nonsyn-sliding.pl* script to compute both metrics in 100-kb nonoverlapping windows.

To assess the prevalence of low-frequency variants in each population, a modified version of $\Delta \Theta_{S}=\;1-(\pi_{S}/\Theta_{S})$, which outperforms Tajima's *D* in detecting excess of low-frequency variants (Qiu et al. 2022), was calculated. As a recently bottlenecked population grows, mutations are initially rare (Tajima 1989a), resulting in higher values of $\Delta \Theta_{S}$. Because most genetic differences among populations occurred in noncoding regions, we calculated a genome-wide version as ΔΘ=1−(π/Θ).

For pairwise *F*_ST_, the alignment files from the four pools were combined to produce a single mpileup file using *SAMtools*. The resulting file was then converted into a synchronized file following *PoPoolation2*'s manual. *F*_ST_ was then calculated using *fst-sliding.pl* available under *PoPoolation2* (Kofler et al. 2011b), in 100-kb nonoverlapping windows.

### Repeat Quantification and TE Insertion Identification

We used *dnaPipeTE* (v.1.3.1) (Goubert et al. 2015) to assemble, annotate, and quantify the repeats in the pools using raw reads from each sample. The *dnaPipeTE* involves multiple steps (see Goubert 2023 for further details). Briefly, the first step is to sample a low representation (often <1× genome coverage) from the input short-read file. Next, the sampled reads are assembled by *Trinity* (Grabherr et al. 2011) into sequences likely originating from repeats in the genome. These sequences are then searched for known repeats using *RepeatMasker*, which can be run using publicly available repeat databases or a de novo TE library specific to the studied species. Finally, an additional short-read sample is blasted against the assembled and annotated repeat sequences to estimate the relative abundance of each repeat in the genome and to compute the TE landscape, which depicts the percentage of divergence between the raw reads and the *dnaPipeTE*-produced repeat sequences.

*dnaPipeTE* was run using the TE library produced above on low coverage read samples (options: -genome_size 172680286 -genome_coverage 0.1) from each population with increasing number of iterations (-sample_number 2 to 5). We then kept the iteration that maximized the N50 of the assembled repeat contigs, although the different iterations produced similar results. The number of iterations that best performed was as follows: three for BH, four for KP, two for MO and RH. *dnaPipeTE* was also used to quantify repeats in Colombian *H. hampei* strain using previously published short raw reads obtained from NCBI (accession numbers SRR11579638 and SRR11579639; Navarro-Escalante et al. 2021).

To estimate TE abundance and frequencies of unique TE insertions in each of the studied populations, *PoPoolationTE2* (Kofler et al. 2016) was used. Briefly, using the TE library and *RepeatMasker*, the reference genome was masked and combined with the TE library to produce a TE-merged reference genome. A TE hierarchy required by *PoPoolationTE2* was generated for every entry in the TE library: its ID, order, and family information. Then paired-end reads from each population were mapped to the TE-merged reference genome with *BWA-BWASW* (Li and Durbin 2009), and paired-end information was recovered with *PoPoolationTE2 se2pe*. The resulting bam files were then used to generate a ppileup (physical pileup) file using *PoPoolationTE2 ppileup* with the --*map-qual 15* option. To account for insert size differences among populations, the physical coverage was subsampled to equal levels (target coverage = 10) in the populations, as recommended by Kofler et al. (2016).

Next, TE insertions and their frequency in each sample were identified separately by running *PoPoolationTE2* with the following parameters: (i) identifySignatures (*--min-count* 2), (ii) frequency, (iii) filterSignatures (*--max-otherte-count* 2 *--max-structvar-count* 2), and (iv) pairupSignatures (*--min-distance* -200 --*max-distance* 300). Lastly, we recovered TE insertions uniquely identified in each population and visualized their frequency distribution in R.

## Results

### Genome Assembly Hham4.1 for *H. hampei*

Using high molecular weight DNA extracted from a pool of 25 female beetles from Hopewell, Jamaica, we generated 9.4 Gb long-read data (Oxford Nanopore MinION) and produced a 172.7 Mb assembly (Hham4.1) for *H. hampei* (supplementary table S2, Supplementary Material online). The Hham4.1 assembly comprises 114 scaffolds containing 98.9% complete BUSCOs (C:98.9%[S:98.4%,D:0.5%],F:0.3%,M:0.8%,n:2124) and is an improvement over previous genome versions with a scaffold N50 of 14 Mb and 96% of the assembly contained within 16 scaffolds (supplementary table S3, Supplementary Material online). Three of the 16 scaffolds (scaffolds 1, 6, and 7) were enriched for Coleopteran telomeric repeats (AACCC, AACCT, and ACCTG) at both ends (supplementary fig. S1, Supplementary Material online), suggesting they likely represent three of the 1n = 7 chromosomes of *H. hampei* (Brun et al. 1995; Constantino et al. 2011; Navarro-Escalante et al. 2021). The remaining scaffolds showed telomeric repeats at only one end (e.g. scaffolds 3, 4, and 8), indicating they likely represent chromosomal arms (supplementary fig. S1, Supplementary Material online), or lacked telomeric repeats altogether, suggesting they are fragments of larger chromosomes (e.g. scaffolds 15 and 16).

The *funannotate* pipeline and publicly available RNA-seq data of male and female beetles (SRA accessions: SRR11858905 and SRR11858906) were used to annotate Hham4.1, yielding 15,899 genes (18,624 transcripts) of which 13,811 were annotated with *InterPro* (Paysan-Lafosse et al. 2023) based on a known protein domain. Assessment of the annotation completeness with *BUSCO* showed that 97.3% of the BUSCOs (C:97.3%[S:96.4%,D:0.9%],F:0.3%,M:2.4%,n:2124) were found to be complete in the annotation.

The total repeat content in the *H. hampei* genome assembly is ∼27% (or 46.43 Mb). While a large proportion (35.7%) of *H. hampei* repeats are unclassified, 42.5% of the identified repeat elements belong to the class II DNA transposons, and 21.8% represent class I retrotransposons with 14.1%, 7.7% LTR, and LINE elements, respectively. The repeat content of the current assembly is substantially higher compared to previous assemblies (∼2.7% to 8%) (Vega et al. 2015; Hernandez-Hernandez et al. 2017), but aligns with the range of estimates observed in other Coleoptera species (Petersen et al. 2019). To investigate whether this increase compared to previous estimates can be explained by the high contiguity of Hham4.1 or reflects population-specific variation, given that earlier assemblies were based on Colombian CBB samples, we used *dnaPipeTE* (Goubert et al. 2015), a reference-free method for exploring mobilomes from raw short reads. The direct estimate from unassembled short reads suggests a higher repeat content in Colombian *H. hampei* (∼28% vs. ∼8% previously reported), similar to our estimates from the Jamaican samples (supplementary table S4, Supplementary Material online). Together, these findings indicate that compared to previous fragmented assemblies, the substantial improvement of Hham4.1 significantly contributed to the identification and annotation of genomic repeats.

**Fig. 1.**
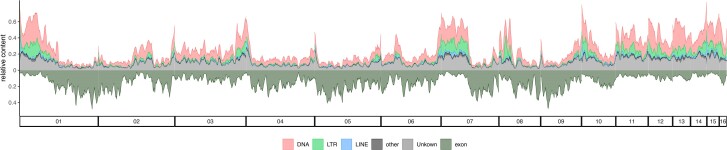
Genome architecture in *H. hampei*. Relative content of TE-derived and exonic sequences across the 16 largest scaffolds of the *H. hampei* genome. Shown are DNA transposons (DNA), long terminal repeat (LTR), long interspersed nuclear element (LINE) retrotransposons, unclassified (Unknown), and other TEs (other).

The genome-wide distribution of repeats in *H. hampei* was heterogenous with regions showing high content of TE-derived sequences (>50%; e.g. scaffolds 1, 7, 12, and 13) and others with reduced TE content (Fig. 1). Exonic content on the other hand was increased in TE-poor regions and was reduced in TE-rich regions of the *H. hampei* genome assembly (Fig. 1), consistent with studies reporting a negative correlation between TE and gene content (Lee and Langley 2010; Fablet et al. 2023).

### Genetic Variation among Jamaican Populations of *H. Hampei*

**Fig. 2.**
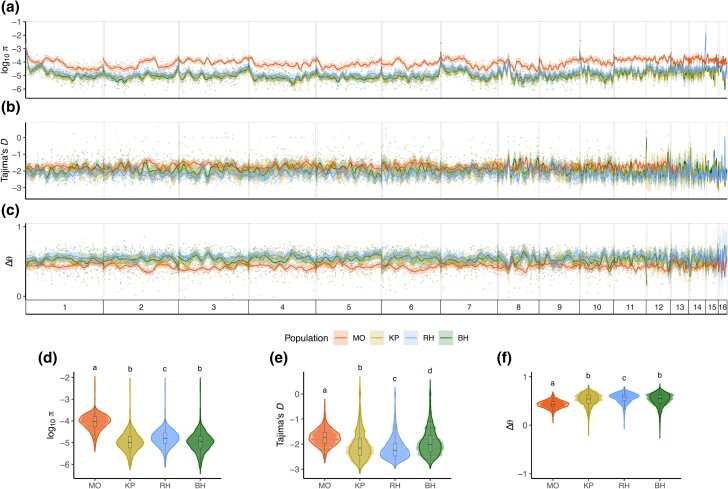
Genome-wide distribution of diversity across the 16 largest scaffolds of the *H. hampei* genome. a and d) Patterns of genome-wide nucleotide diversity; b and e) Tajima's *D* estimates; and c and f) ΔΘ estimates in four CBB populations from MO in the Highlands, KP in the Lowlands, RH in the Blue Mountains, and BH in the Highlands of Jamaica. Different lowercase letters represent significant differences according to pairwise Wilcoxon rank sum post hoc tests.

We used pool-seq to characterize patterns of genome-wide nucleotide diversity (*π*) within populations collected across Jamaica (Fig. 2a and d). Estimates of *π* calculated in 100 kb nonoverlapping windows varied significantly among the four populations (Fig. 2d; Kruskal–Wallis rank sum test, $\chi^{2}$ = 2,963, df = 3, *P* < 2.2e^−16^) with MO showing a 3fold higher average genome-wide genetic variation (*π* = 1.3e^−4^) compared to the other populations (*π* = 3.79e^−5^ in KP; *π* = 3.86e^−5^ in BH; *π* = 4.36e^−5^ in RH). This variation was only marginal when considering variation at synonymous and nonsynonymous sites (supplementary table S5, Supplementary Material online), suggesting that in the MO population, variation accumulated essentially in noncoding genomic regions.

### Population Bottleneck and History of Jamaican CBBs

To explain the genetic patterns observed in the MO population (i.e. increased levels of nucleotide diversity), we hypothesized that its introduction event predates those of KP, BH, and RH. To investigate this, we analyzed deviations from neutrality using Tajima's *D*, a widely used metric in population genetics for inferring demographic processes such as bottlenecks (Tajima 1989a, 1989b). Additionally, a modified version of $\Delta \Theta_{S}=\;1-(\pi_{S}/\Theta_{S})$, a measure sensitive to excess rare variants in expanding populations following strong bottlenecks (Qiu et al. 2022), was calculated. However, since most genetic differences occur in noncoding genome regions, we calculated a genome-wide version instead as ΔΘ=1−(π/Θ). Similar to Tajima's *D*, this metric also relies on *π*, and its expected value Θ can detect excess of rare variants typically found in expanding populations after a strong bottleneck (Tajima 1989b). Following a strong genetic bottleneck, e.g. due to founder effects, genetic variation is rare, resulting in a strongly negative Tajima's *D* and a ΔΘ approaching 1 (Qiu et al. 2022). However, as the population grows and mutations increase in frequency, Tajima's *D* increases while ΔΘ decreases toward zero (Qiu et al. 2022).

Similar to *π*, Tajima's *D* estimates varied across populations (Fig. 2b and e; Kruskal–Wallis rank sum test, $\chi^{2}$ = 961.37, df = 3, *P* < 2.2e^−16^) and were significantly higher in the MO population (Tajima's *D* = −1.74) compared to the other populations (Tajima's *D* = −2 in KP; Tajima's *D* = −1.92 in BH; Tajima's *D* = −2.16 in RH). In contrast, ΔΘ estimates were significantly reduced in the MO population (ΔΘ = 0.43) relative to the other populations (ΔΘ = 0.52 in KP; ΔΘ = 0.52 in BH; ΔΘ = 0.56 in RH) (Fig. 2c and f; Kruskal–Wallis rank sum test, $\chi^{2}$ = 1,376.6, df = 3, *P* < 2.2e^−16^).

**Fig. 3.**
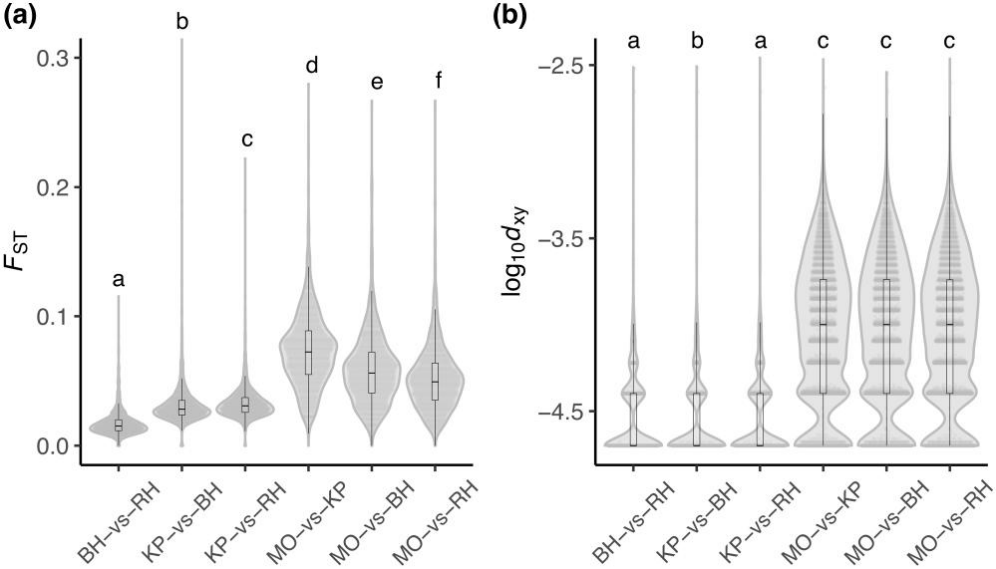
Violin plots showing a) genetic differentiation and b) absolute divergence among the four Jamaican populations: MO in the Highlands, KP in the Lowlands, RH in the Blue Mountains, and BH in the Highlands of Jamaica. Different lowercase letters represent significant differences according to pairwise Wilcoxon rank sum post hoc tests.

The presence of two or more genetically divergent lineages is an indication that these populations likely originated from distinct genetic sources. To investigate this, we estimated the levels of genetic differentiation among the four populations. Average pairwise genetic differentiation *F*_ST_ among the studied populations was reduced and varied between 0.017 (BH vs. RH) and 0.073 (MO vs. KP), with the highest estimates observed in pairwise comparisons involving the MO population (Fig. 3a; Kruskal–Wallis rank sum test, $\chi^{2}$ = 5,607.4, df = 5, *P* < 2.2e^−16^).

**Fig. 4.**
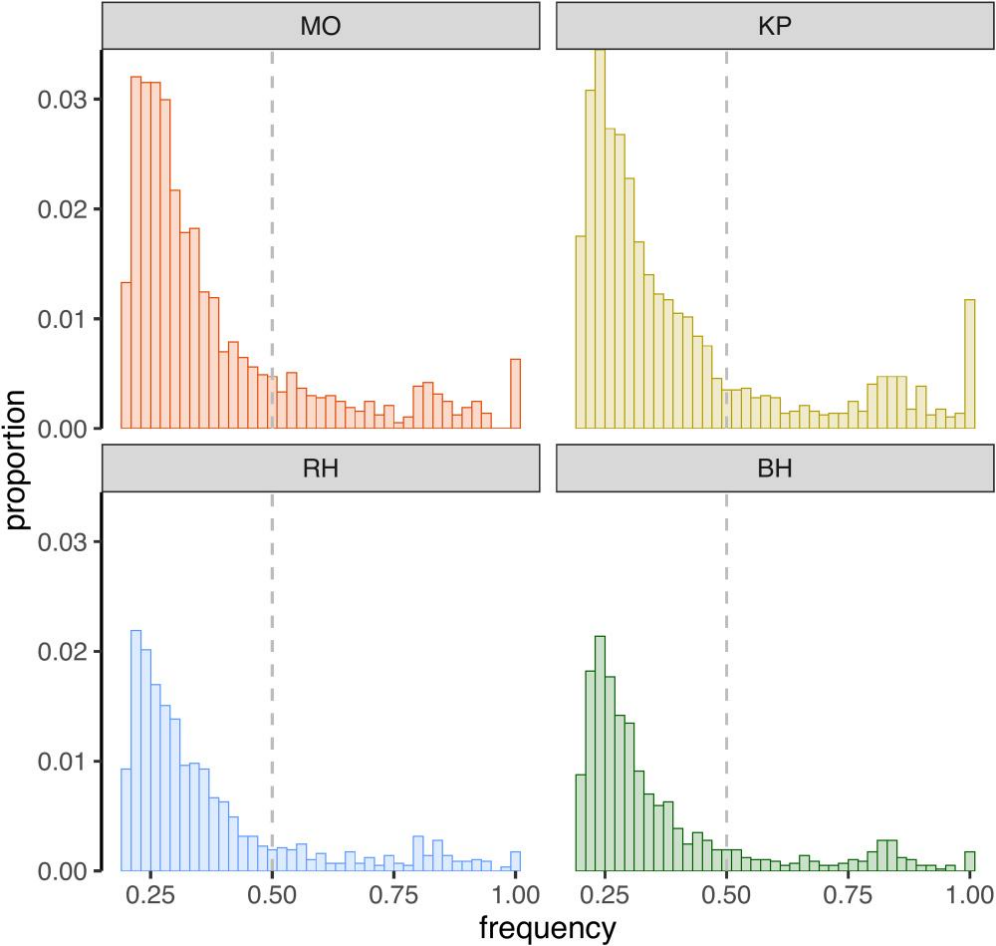
TE frequency distribution across the four Jamaican populations of *H. hampei* from the Highlands (MO and BH), Lowlands (KP), and Blue Mountains (RH). Gray dashed line splits the distribution into low- (<0.5) and high- (>0.5) frequency TE insertions.

However, because *F*_ST_ is sensitive to within-population levels of genetic variation, absolute divergence (*d*_XY_), a measure that is independent of genetic variation within each population, was also calculated by sampling and comparing the most abundant allele(s) in each population. Similar to *F*_ST_, pairs involving MO displayed significantly higher divergence compared to the other pairs (Fig. 4b; Kruskal–Wallis rank sum test, $\chi^{2}$ = 1,2467, df = 5, *P* < 2.2e^−16^). We found no significant differences between pairs involving the MO population, suggesting equal genetic distance between MO and the three other populations. Moreover, only subtle differences were observed within pairs involving BH, KP, and RH (supplementary table S6, Supplementary Material online). Together, these results suggest two distinct genetic lineages among Jamaican CBB populations: one leading to MO, divergent from all other populations, and another leading to BH, KP, and RH.

### Surge in TE Activity in *H. hampei* Jamaican Populations

As a result of changing environmental conditions, introduced populations of invasive species can exhibit high activity of TEs (Casacuberta and González 2013; Stapley et al. 2015). Such activity of TEs is expected to result in a high prevalence of low-frequency TEs as well as a high frequency of almost identical TE copies within a population. This is because novel TE insertions face strong selective pressure and do not have enough time to increase in frequency or to diverge by accumulating mutations (Lerat et al. 2019). To investigate this, we identified unique TE insertions in each population using *PoPoolationTE2* (Kofler et al. 2016). The MO and KP populations displayed the highest numbers of unique TE insertions (1,817 and 1,889, respectively), whereas BH and RH had comparatively lower numbers (942 and 1,063, respectively). The TE frequency distribution across all populations was skewed toward low-frequency TEs (Fig. 4), indicating a recent surge in TE activity in the Jamaican *H. hampei* populations. In MO and KP, we found increased proportions of fixed TE insertions (allele frequency = 1), potentially reflecting examples of adaptive TE insertions.

**Fig. 5.**
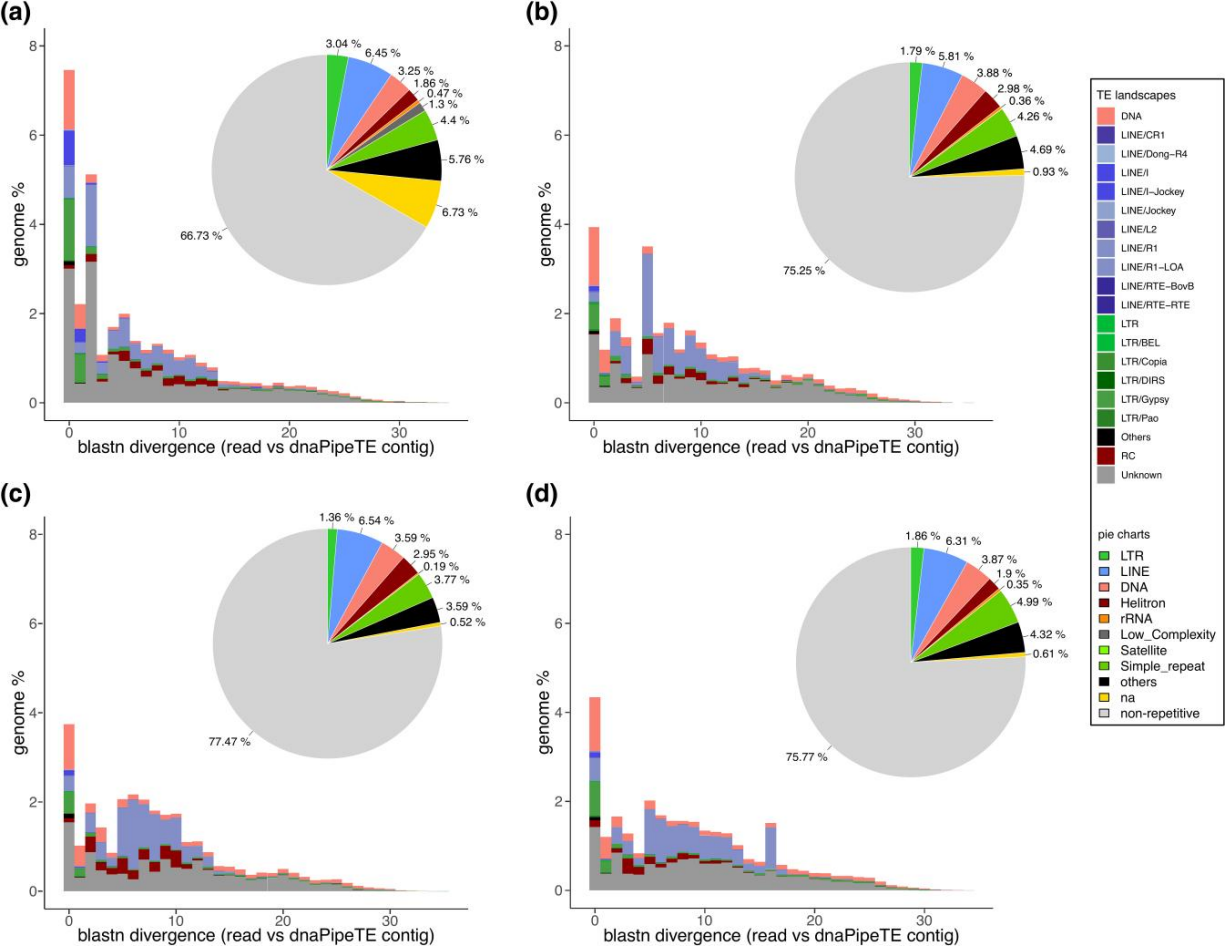
Relative genome proportions (pie charts) and TE landscapes (bar plots) of the main repeat families found in populations of *H. hampei* from MO a) in the Highlands, KP b) in the Lowlands, RH c) in the Blue Mountains, and BH d) in the Highlands of Jamaica. *dnaPipeTE* with 0.1× coverage per sample was used to explore signatures of TE activity. Note that blastn divergence refers to the percentage of divergence between the raw short reads and the *dnaPipeTE*-assembled and annotated repeat sequences, with each bin on the *x* axis representing 1% divergence.

Signatures of recent TE activity in the Jamaican CBB populations were further explored using *dnaPipeTE* (Goubert et al. 2015). The total genomic coverage of repeats varied, approximately constituting ∼25% in KP, BH, and RH, and ∼33% in MO (Fig. 5; pie charts); these estimates are in line with estimates based on the genome assembly (∼27%).

The MO population exhibited a higher proportion of unclassified repeats (6%) compared to other populations (<1%), while the proportions of other major repeat classes (e.g. LINE, DNA, and Satellite) were similar. Divergence-based TE landscape plots generated with *dnaPipeTE* showed that the prevalence of nearly identical TE copies (e.g. low blastn divergence), particularly of LINE/R1 and LTR/Gypsy but also unclassified elements, varied between populations (Fig. 5; TE landscapes). In MO, recent TE insertions accounted for almost 8% of the genome. In the other populations, they account for only 4% or less. Taken together, these results indicate a recent surge in TE activity (Casacuberta and González 2013; Stapley et al. 2015), particularly involving LINE/R1 and LTR/Gypsy elements within introduced CBB populations in Jamaica.

## Discussion

Tramp species spread through human activities and successfully establish populations, despite the expected negative effects of passing through a genetic bottleneck. These negative effects include the loss of heterozygote advantage of some loci and/or increasing genetic load of others, and a reduction in adaptive potential (Schrieber and Lachmuth 2017). *Hypothenemus hampei* is highly specialized and spends most of its life sheltered inside a coffee berry (Lee et al. 2023), which likely helps the beetle mitigate this paradox. Consequently, the necessity for immediate adaptation to novel conditions outside the coffee berry habitat is low. Despite this apparent ecological niche specialization, CBB beetles exhibit a remarkable adaptive capacity, demonstrated by the repeated evolution of pesticide resistance (Brun et al. 1989; Olivier Brun and Maxwell Suckling 1992; ffrench-Constant et al. 1994; Witter-Newell 2008; Davi Júnior et al. 2021).

Using a hybrid assembly strategy integrating long and short reads, we produced a high-quality genome assembly for *H. hampei*, which represents the smallest bark beetle genome out of the 297 coleopterans documented in the Animal Genome Size Database (Gregory 2024). Only two other beetle species reported in this database have a similarly small genome (*Tribolium audax* and *Tribolium destructor*) (Alvarez-Fuster et al. 1991). The new assembly (Hham4.1) spans 172.7 Mb and is significantly more contiguous compared to previously published genomes. The scaffold N50 of Hham4.1 stands at 14 Mb compared to 0.044 to 0.34 Mb in previous assemblies (Vega et al. 2015; Navarro-Escalante et al. 2021), with 96% of the assembly contained within the largest 16 scaffolds. At least three of these scaffolds likely represent three of the 1n = 7 chromosomes of *H. hampei* (Brun et al. 1995; Constantino et al. 2011; Navarro-Escalante et al. 2021), while the remaining scaffolds probably correspond to chromosomal arms. Although the current assembly shows significant improvement over previous versions, future studies using e.g. Hi-C contact maps are necessary to refine it to chromosome level.

The repeat content of the current assembly of ∼27% is substantially higher than that reported for previous assemblies (∼2.7% to 8%) (Vega et al. 2015; Hernandez-Hernandez et al. 2017), but aligns with the range of estimates observed in other Coleoptera species (Petersen et al. 2019). This increase compared to previous estimates is likely explained by the substantially increased contiguity of Hham4.1, facilitating genomic repeat identification and annotation, including 687 de novo repeats generated from the current genome assembly. Investigation of the raw unassembled short reads previously used to generate a reference genome for *H. hampei* showed that the repeat content is much higher than previously reported. Apart from methodological reasons, differences in repeat content between this and previous assemblies may to some extent also reflect population-specific variation, as earlier assemblies were based on Colombian CBB samples. A substantial proportion (35.7%) of repetitive elements in *H. hampei* remained unclassified, while 42.5% were classified as class II DNA transposons and 21.8% represented class I retrotransposons. The prevalence of unclassified elements fits observations in other Coleoptera species (Petersen et al. 2019).

Amplified fragment length polymorphism markers indicate low levels of genetic variability for introduced CBB populations from Brazil (Gil et al. 2015b). Jamaica, with its recent and traceable history of CBB introduction, offers an opportunity to study how genetic variation changes over time and space. Using the newly assembled genome, first analyses of pool-seq data from four populations across Jamaica revealed an overall low level of genetic diversity, as indicated by low nucleotide diversity (*π*) and Tajima's *D*, consistent with the recent introduction of the beetle to Jamaica (Reid 1983). Genetic diversity appeared more pronounced in the MO population, with both *π* and Tajima's *D* estimates being higher compared to the KP, BH, and RH populations. This is primarily driven by an accumulation of neutral variation, particularly in noncoding regions, within the MO population. Absolute and relative genetic differentiation analyses revealed two relatively distinct lineages: one leading to MO and another leading to BH, KP, and RH.

Three plausible scenarios arise from these findings. First, MO and the other Jamaican populations together represent two independent introduction events from different source populations and the KP, RH and BH lineage has spread more on the island than MO. Based on previous studies, the potential source populations for these introduction events could be either from Africa/Asia (Andreev et al. 1998; Gauthier 2010) or from the Americas, particularly Brazil (Benavides et al. 2005). Alternatively, all populations stem from one source population, with elevated standing genetic variation leading to separate lineages of the MO population and the others. Finally, the CBB was introduced to Jamaica only once, and the MO population is the oldest on the island, thus having had more time to accumulate genetic variation and divergence, and from here it spread to the three younger populations. The observation that allele frequencies are less skewed toward rare alleles (i.e. higher Tajima's *D* and reduced ΔΘ) in MO favors a scenario that the MO introduction occurred much earlier. However, whether the three populations KP, RH, and BH all originated from the MO population or shared the same or different source populations remains unclear. Future investigations should include samples from the CBB's native range in Africa, as well as from South and Central America to resolve the invasion history of CBB in Jamaica.

TEs, which affect various aspects of genome dynamics, can become active under stressful conditions (Horváth et al. 2017). For tramp species that routinely encounter novel and potentially stressful conditions, such TE activity can prove advantageous, generating genetic variation and facilitating adaptation (Capy et al. 2000; Stapley et al. 2015; Schrader and Schmitz 2019). The analysis of TE dynamics revealed an increase in low-frequency TEs as well as TEs with reduced divergence, suggesting a recent surge in TE activity, particularly involving LINE/R1 and LTR/Gypsy elements, within Jamaican CBBs. This pattern of recent and prominent proliferation of LTR/Gypsy has also been observed in introduced populations of the ant species *Cardiocondyla obscurior* (Errbii et al. 2021) but distinguishes *H. hampei* from other Coleopteran genomes analyzed so far, with the exception of *T. castaneum* (Petersen et al. 2019). However, it will be necessary to study how TE dynamics and frequencies vary over time by analyzing samples from the same locations in the future.

How the increased activity of TEs affects the CBB genome is unclear. TE activity is mutagenic (Bourque et al. 2018), capable of inducing structural variants with significant effects at both the genomic and phenotypic levels (Schrader and Schmitz 2019). While most of these TE-induced mutations are expected to be deleterious or neutral, they can also give rise to adaptive variants as shown in flies (González and Petrov 2009; González et al. 2010), butterflies (Casacuberta and González 2013; Woronik et al. 2019), plants (Li et al. 2018; Niu et al. 2019), and humans (Xia et al. 2024).

The recent and well-documented introduction of the CBB *H. hampei* to Jamaica presents an excellent opportunity to investigate rapid evolutionary processes in this highly successful pest species. The present study offers first insights into population genomic dynamics, particularly the activity of TEs. For future studies, the newly generated high-quality genome provides the basis for in-depth exploration of genetic bottlenecks, genetic drift, and their implications for the biology of this specialized and devastating pest.

## Supplementary Material

evae217_Supplementary_Data

## Data Availability

The genome assembly and annotation as well as the raw sequencing data are available at the NCBI BioProject database (https://www.ncbi.nlm.nih.gov/bioproject/) under accession number PRJNA1110552. This study used scripts from previous bioinformatic pipeline that can be found at https://github.com/merrbii/CobsPopGenomics. The scripts for assembling and annotating the CBB reference genome can be found at https://zivgitlab.uni-muenster.de/schradel/CBB.

## References

[evae217-B1] Allaby M . A dictionary of zoology. UK: Oxford University Press; 2009 [accessed 2024 Apr 25]. https://www.oxfordreference.com/display/10.1093/acref/9780199233410.001.0001/acref-9780199233410.

[evae217-B2] Alvarez-Fuster A, Juan C, Petitpierre E. Genome size in *Tribolium* flour-beetles: inter- and intraspecific variation. Genet Res. 1991:58(1):1–5. 10.1017/S0016672300029542.

[evae217-B3] Andreev D, Breilid H, Kirkendall L, Brun LO, Ffrench-Constant RH. Lack of nucleotide variability in a beetle pest with extreme inbreeding. Insect Mol Biol. 1998:7(2):197–200. 10.1046/j.1365-2583.1998.72064.x. 9535165

[evae217-B4] Benavides P, Vega FE, Romero-Severson J, Bustillo AE, Stuart JJ. Biodiversity and biogeography of an important inbred pest of coffee, coffee berry borer (Coleoptera: Curculionidae: Scolytinae). Ann Entomol Soc Am. 2005:98(3):359–366. 10.1603/0013-8746(2005)098[0359:BABOAI]2.0.CO;2.

[evae217-B5] Bolger AM, Lohse M, Usadel B. Trimmomatic: a flexible trimmer for Illumina sequence data. Bioinformatics. 2014:30(15):2114–2120. 10.1093/bioinformatics/btu170. 24695404 PMC4103590

[evae217-B6] Bourque G, Burns KH, Gehring M, Gorbunova V, Seluanov A, Hammell M, Imbeault M, Izsvák Z, Levin HL, Macfarlan TS, et al Ten things you should know about transposable elements. Genome Biol. 2018:19(1):199. 10.1186/s13059-018-1577-z. 30454069 PMC6240941

[evae217-B7] Brown M, Gonzlez De la Rosa PM, Mark B. 2023. A telomere identification toolkit. [Computer software]. Software v0.2.41. 10.5281/zenodo.10091385.

[evae217-B8] Brun L, Marcillaud C, Gaudichon V, Suckling D. Endosulfan resistance in *Hypothenemus hampei* (Coleoptera: Scolytidae) in New Caledonia. J Econ Entomol. 1989:82(5):1331–1316. Publisher: Entomological Society of America. 10.1093/jee/82.5.1311.

[evae217-B9] Brun LO, Stuart J, Gaudichon V, Aronstein K, French-Constant RH. Functional haplodiploidy: a mechanism for the spread of insecticide resistance in an important international insect pest. Proc Natl Acad Sci U S A. 1995:92(21):9861–9865. 10.1073/pnas.92.21.9861. 7568233 PMC40902

[evae217-B10] Cantalapiedra CP, Hernández-Plaza A, Letunic I, Bork P, Huerta-Cepas J. eggNOG-mapper v2: functional annotation, orthology assignments, and domain prediction at the metagenomic scale. Mol Biol Evol. 2021:38(12):5825–5829. 10.1093/molbev/msab293. 34597405 PMC8662613

[evae217-B11] Capy P, Gasperi G, Biémont C, Bazin C. Stress and transposable elements: co-evolution or useful parasites? Heredity (Edinb). 2000:85(2):101–106. 10.1046/j.1365-2540.2000.00751.x. 11012710

[evae217-B12] Casacuberta E, González J. The impact of transposable elements in environmental adaptation. Mol Ecol. 2013:22(6):1503–1517. 10.1111/mec.12170. 23293987

[evae217-B13] Chapman EG, Messing RH, Harwood JD. Determining the origin of the coffee berry borer invasion of Hawaii. Ann Entomol Soc Am. 2015:108(4):585–592. 10.1093/aesa/sav024.

[evae217-B14] Cole E, Keller RP, Garbach K. Risk of invasive species spread by recreational boaters remains high despite widespread adoption of conservation behaviors. J Environ Manage. 2019:229:112–119. 10.1016/j.jenvman.2018.06.078. 30449301

[evae217-B15] Constantino LM, Navarro L, Berrio A, Acevedo Fe, Rubio D, Benavides P. Aspectos biológicos, morfológicos y genéticos de *Hypothenemus obscurus* e *Hypothenemus hampei* (Coleoptera: Curculionidae: Scolytinae). Revista Colomb Entomol. 2011:37(2):173–182. 10.25100/socolen.v37i2.9071.

[evae217-B16] Davi Júnior SD, Soares WS, Celoto FJ, Fernandes FL, Oliveira MM, Botrel GB. Resistance and effect of insecticide-treated coffee berries of different varieties to the penetration of *Hypothenemus hampei* (Coleoptera: Curculionidae: Scolytinae). Coffee Sci. 2021:16:e161874–e161874. 10.25186/.v16i.1874. ISSN 1984-3909

[evae217-B17] Dobin A, Davis CA, Schlesinger F, Drenkow J, Zaleski C, Jha S, Batut P, Chaisson M, Gingeras TR. STAR: ultrafast universal RNA-seq aligner. Bioinformatics. 2013:29(1):15–21. 10.1093/bioinformatics/bts635. 23104886 PMC3530905

[evae217-B18] Errbii M, Ernst UR, Lajmi A, Privman E, Gadau J, Schrader L. Evolutionary genomics of socially polymorphic populations of *Pogonomyrmex californicus*. BMC Biol. 2024:22(1):109. 10.1186/s12915-024-01907-z. 38735942 PMC11089791

[evae217-B19] Errbii M, Keilwagen J, Hoff KJ, Steffen R, Altmüller J, Oettler J, Schrader L. Transposable elements and introgression introduce genetic variation in the invasive ant *Cardiocondyla obscurior*. In: Molecular ecology. Vol. 30. USA: John Wiley & Sons, Ltd; 2021. p. 6211–6228. 10.1111/mec.16099 34324751

[evae217-B20] Fablet M, Salces-Ortiz J, Jacquet A, Menezes BF, Dechaud C, Veber P, Rebollo R, Vieira C. A quantitative, genome-wide analysis in *Drosophila* reveals transposable elements’ influence on gene expression is species-specific. Genome Biol Evol. 2023:15(9):evad160. 10.1093/gbe/evad160. 37652057 PMC10492446

[evae217-B21] ffrench-Constant R, Steichen JC, Brun LO. A molecular diagnostic for endosulfan insecticide resistance in the coffee berry borer *Hypothenemus hampei* (Coleoptera: Scolytidae). Bull Entomol Res. 1994:84(1):11–15. 10.1017/S000748530003217X.

[evae217-B22] Flynn JM, Hubley R, Goubert C, Rosen J, Clark AG, Feschotte C, Smit AF. RepeatModeler2 for automated genomic discovery of transposable element families. Proc Natl Acad Sci U S A. 2020:117(17):9451–9457. 10.1073/pnas.1921046117. 32300014 PMC7196820

[evae217-B23] Frankham R . Resolving the genetic paradox in invasive species. Heredity (Edinb). 2005:94(4):385–385. 10.1038/sj.hdy.6800634. 15602569

[evae217-B24] Frankham R, Lees K, Montgomery ME, England PR, Lowe EH, Briscoe DA. Do population size bottlenecks reduce evolutionary potential? Anim Conserv. 1999:2(4):255–260. 10.1111/j.1469-1795.1999.tb00071.x.

[evae217-B25] Gauthier N . Multiple cryptic genetic units in *Hypothenemus hampei* (Coleoptera: Scolytinae): evidence from microsatellite and mitochondrial DNA sequence data. Biol J Linn Soc Lond. 2010:101(1):113–129. 10.1111/j.1095-8312.2010.01483.x.

[evae217-B26] Gil ZN, Benavides P, De Souza O, Acevedo FE, Lima E. Molecular markers as a method to evaluate the movement of *Hypothenemus hampei* (Ferrari). J Insect Sci. 2015a:15(1):72. 10.1093/jisesa/iev058. 26078300 PMC4677496

[evae217-B27] Gil ZN, Benavides P, Hernández E, Nogueira K, Fontes D, Lima E. Genetic variability and population structure of the coffee berry borer *Hypothenemus hampei* Ferrari in Brazil inferred by AFLP markers. 2015b [accessed 2023 Jun 22]. https://biblioteca.cenicafe.org/handle/10778/548.

[evae217-B28] Giraldo-Jaramillo M, Garcia AG, Parra JR. Biology,thermal requirements, and estimation of the number of generations of *Hypothenemus hampei* (Ferrari, 1867) (Coleoptera: Curculionidae) in the state of São Paulo, Brazil. J Econ Entomol. 2018:111:2192–2200. 10.1093/jee/toy162. 29947807

[evae217-B29] González J, Karasov TL, Messer PW, Petrov DA. Genome-wide patterns of adaptation to temperate environments associated with transposable elements in *Drosophila*. PLoS Genet. 2010:6(4):e1000905. 10.1371/journal.pgen.1000905. 20386746 PMC2851572

[evae217-B30] González J, Petrov DA. The adaptive role of transposable elements in the *Drosophila* genome. Gene. 2009:448(2):124–133. 10.1016/j.gene.2009.06.008. 19555747 PMC2784284

[evae217-B31] Goubert C . Assembly-free detection and quantification of transposable elements with dnaPipeTE. Methods Mol Biol. 2023:2607:25–43. 10.1007/978-1-0716-2883-6_2. 36449156

[evae217-B32] Goubert C, Modolo L, Vieira C, ValienteMoro C, Mavingui P, Boulesteix M. De novo assembly and annotation of the Asian tiger mosquito (*Aedesalbopictus*) repeatome with dnaPipeTE from raw genomic reads and comparative analysis with the yellow fever mosquito (*Aedes aegypti*). Genome Biol Evol. 2015:7(4):1192–1205. 10.1093/gbe/evv050. 25767248 PMC4419797

[evae217-B33] Grabherr MG, Haas BJ, Yassour M, Levin JZ, Thompson DA, Amit I, Adiconis X, Fan L, Raychowdhury R, Zeng Q, et al Full-length transcriptome assembly from RNA-Seq data without a reference genome. Nat Biotechnol. 2011:29(7):644–652. 10.1038/nbt.1883. 21572440 PMC3571712

[evae217-B34] Gregory TR. 2024. Animal Genome Size Database. http://www.genomesize.com.

[evae217-B35] Hamilton LJ, Manoukis NC, Follett PA, Johnson MA. Coffee berry borer (*Hypothenemus hampei*) (Coleoptera: Curculionidae) development across an elevational gradient on Hawai‘i Island: applying laboratory degree-day predictions to natural field populations. PLoS One. 2019:14(7):e0218321. 10.1371/journal.pone.0218321. 31314766 PMC6636796

[evae217-B36] Hernandez-Hernandez EM, Fernández-Medina RD, Navarro-Escalante L, Nuñez J, Benavides-Machado P, Carareto CMA. Genome-wide analysis of transposable elements in the coffee berry borer *Hypothenemus hampei* (Coleoptera: Curculionidae): description of novel families. Mol Genet Genomics. 2017:292(3):565–583. 10.1007/s00438-017-1291-7. 28204924

[evae217-B37] Horváth V, Merenciano M, González J. Revisiting the relationship between transposable elements and the eukaryotic stress response. Trends Genet. 2017:33(11):832–841. 10.1016/j.tig.2017.08.007. 28947157

[evae217-B38] Hu J, Wang Z, Liang F, Liu S-L, Ye K, Wang D-P. NextPolish2: a repeat-aware polishing tool for genomes assembled using HiFi long reads. Genom Proteom Bioinform. 2024:22(1):qzad009. 10.1093/gpbjnl/qzad009. PMC12016036 38862426

[evae217-B39] Infante F . Pest management strategies against the coffee berry borer (Coleoptera: Curculionidae: Scolytinae). J Agric Food Chem. 2018:66(21):5275–5280. 10.1021/acs.jafc.7b04875. 29528640

[evae217-B40] Infante F, Jaramillo J, Castillo A, Vega F. The coffee berry borer, *Hypothenemus hampei* (Ferrari) (Coleoptera: Curculionidae): a short review, with recent findings and future research directions. Terr Arthropod Rev. 2009:2(2):129–147. 10.1163/187498209X12525675906031.

[evae217-B41] Jaramillo J, Muchugu E, Vega FE, Davis A, Borgemeister C, Chabi-Olaye A. Some like it hot: the influence and implications of climate change on coffee berry borer (*Hypothenemus hampei*) and coffee production in East Africa. PLoS One. 2011:6(9):e24528. 10.1371/journal.pone.0024528. 21935419 PMC3173381

[evae217-B42] Jarju LB, Fillinger U, Green C, Louca V, Majambere S, Lindsay SW. Agriculture and the promotion of insect pests: rice cultivation in river floodplains and malaria vectors in The Gambia. Malar J. 2009:8(1):170. 10.1186/1475-2875-8-170. 19635125 PMC2734858

[evae217-B43] Johnson MA, Manoukis NC. Abundance of coffee berry borer in feral, abandoned and managed coffee on Hawaii Island. J Appl Entomol. 2020:144(10):920–928. 10.1111/jen.12804.

[evae217-B44] Johnson MA, Ruiz-Diaz CP, Manoukis NC, Verle Rodrigues JC. Coffee berry borer (*Hypothenemus hampei*), a global pest of coffee: perspectives from historical and recent invasions, and future priorities. Insects. 2020:11(12):882. 10.3390/insects11120882. 33322763 PMC7763606

[evae217-B45] Jones P, Binns D, Chang H-Y, Fraser M, Li W, McAnulla C, McWilliam H, Maslen J, Mitchell A, Nuka G, et al InterProScan 5: genome-scale protein function classification. Bioinformatics. 2014:30(9):1236–1240. 10.1093/bioinformatics/btu031. 24451626 PMC3998142

[evae217-B46] Keilwagen J, Hartung F, Grau J. Gemoma: homology-based gene prediction utilizing intron position conservation and RNA-seq data. Methods Mol Biol. 2019:1962:161–177. 10.1007/978-1-4939-9173-0_9. 31020559

[evae217-B47] Kofler R, Gómez-Sánchez D, Schlötterer C. PoPoolationTE2: comparative population genomics of transposable elements using pool-seq. Mol Biol Evol. 2016:33(10):2759–2764. 10.1093/molbev/msw137. 27486221 PMC5026257

[evae217-B48] Kofler R, Orozco-terWengel P, De Maio N, Pandey RV, Nolte V, Futschik A, Kosiol C, Schlötterer C. Popoolation: a toolbox for population genetic analysis of next generation sequencing data from pooled individuals. PLoS One. 2011a:6(1):15925. 10.1371/journal.pone.0015925. PMC3017084 21253599

[evae217-B49] Kofler R, Pandey RV, Schlötterer C. Popoolation2: identifying differentiation between populations using sequencing of pooled DNA samples (pool-seq). Bioinformatics. 2011b:27(24):3435–3436. 10.1093/bioinformatics/btr589. 22025480 PMC3232374

[evae217-B50] Laetsch DR, Blaxter ML. BlobTools: interrogation of genome assemblies. F1000Res. 2017:6:1287. 10.12688/f1000research.12232.1.

[evae217-B51] Lee D, Johnson MA, Aristizábal LF, Shriner S, Chan C, Miyasaka S, Wall M. Economic benefits from managing coffee berry borer (*Hypothenemus hampei*) in Hawaii. Insects. 2023:14(4):350. 10.3390/insects14040350. 37103165 PMC10143774

[evae217-B52] Lee YCG, Langley CH. Transposable elements in natural populations of *Drosophila melanogaster*. Philos Trans R Soc Lond B Biol Sci. 2010:365(1544):1219–1228. 10.1098/rstb.2009.0318. 20308097 PMC2871824

[evae217-B53] Lerat E, Goubert C, Guirao-Rico S, Merenciano M, Dufour AB, Vieira C, González J. Population-specific dynamics and selection patterns of transposable element insertions in European natural populations. Mol Ecol. 2019:28(6):1506–1522. 10.1111/mec.14963. 30506554 PMC6849870

[evae217-B54] Li H, Durbin R. Fast and accurate short read alignment with Burrows-Wheeler transform. Bioinformatics. 2009:25(14):1754–1760. 10.1093/bioinformatics/btp324. 19451168 PMC2705234

[evae217-B55] Li H, Handsaker B, Wysoker A, Fennell T, Ruan J, Homer N, Marth G, Abecasis G, Durbin R. The Sequence Alignment/Map format and SAMtools. Bioinformatics. 2009:25(16):2078–2079. 10.1093/bioinformatics/btp352. 19505943 PMC2723002

[evae217-B56] Li Z-W, Hou X-H, Chen J-F, Xu Y-C, Wu Q, González J, Guo Y-L. Transposable elements contribute to the adaptation of *Arabidopsis thaliana*. Genome Biol Evol. 2018:10(8):2140 2150. 10.1093/gbe/evy171. 30102348 PMC6117151

[evae217-B57] Lin Y, Yuan J, Kolmogorov M, Shen MW, Chaisson M, Pevzner PA. Assembly of long error-prone reads using de Bruijn graphs. Proc Natl Acad Sci. 2016:113(52):E8396–E8405. 10.1073/pnas.1604560113. 27956617 PMC5206522

[evae217-B58] Ludwig A, Pippel M, Myers G, Hiller M. DENTIST—using long reads for closing assembly gaps at high accuracy. GigaScience. 2022:11:giab100. 10.1093/gigascience/giab100. 35077539 PMC8848313

[evae217-B59] Miller SA, Dykes DD, Polesky HF. A simple salting out procedure for extracting DNA from human nucleated cells. Nucleic Acids Res. 1988:16(3):1215. 10.1093/nar/16.3.1215. 3344216 PMC334765

[evae217-B60] Miller SE, Sheehan MJ. Sex differences in deleterious genetic variants in a haplodiploid social insect. Mol Ecol. 2023:32(16):4546–4556. 10.1111/mec.17057. 37350360 PMC10528523

[evae217-B61] Mistry J, Chuguransky S, Williams L, Qureshi M, Salazar GA, Sonnhammer ELL, Tosatto SCE, Paladin L, Raj S, Richardson LJ, et al Pfam: the protein families database in 2021. Nucleic Acids Res. 2021:49(D1):D412–D419. 10.1093/nar/gkaa913. 33125078 PMC7779014

[evae217-B62] Moreno-Ramirez N, Bianchi FJJA, Manzano MR, Dicke M. Ecology and management of the coffee berry borer (*Hypothenemus hampei*): the potential of biological control. BioControl. 2024:69(2):199–214. 10.1007/s10526-024-10253-6.

[evae217-B63] Myrie A, Hall T, Luke D, Chinthapalli BR, Tennant P, Robinson D. Coffee berry borer, *Hypothenemus hampei* (Ferrari) (Coleoptera: Curculionidae): activity and infestation in the high mountain and Blue Mountain regions of Jamaica. Insects. 2023:14(8):694. 10.3390/insects14080694. 37623404 PMC10455829

[evae217-B64] Navarro-Escalante L, Hernandez-Hernandez EM, Nuñez J, Acevedo FE, Berrio A, Constantino LM, Padilla-Hurtado BE, Molina D, Gongora C, Acuña R, et al A coffee berry borer (*Hypothenemus hampei*) genome assembly reveals a reduced chemosensory receptor gene repertoire and male-specific genome sequences. Sci Rep. 2021:11(1):4900. 10.1038/s41598-021-84068-1. 33649370 PMC7921381

[evae217-B65] Neph S, Kuehn MS, Reynolds AP, Haugen E, Thurman RE, Johnson AK, Rynes E, Maurano MT, Vierstra J, Thomas S, et al BEDOPS: high-performance genomic feature operations. Bioinformatics. 2012:28(14):1919–1920. 10.1093/bioinformatics/bts277. 22576172 PMC3389768

[evae217-B66] Niu X-M, Xu YC, Li ZW, Bian YT, Hou XH, Chen JF, Zou YP, Jiang J, Wu Q, Ge S, et al Transposable elements drive rapid phenotypic variation in *Capsella rubella*. Proc Natl Acad Sci. 2019:116(14):6908–6913. 10.1073/pnas.1811498116. 30877258 PMC6452725

[evae217-B67] Okonechnikov K, Conesa A, García-Alcalde F. Qualimap 2: advanced multi-sample quality control for high-throughput sequencing data. Bioinformatics. 2016:32(2):292–294. 10.1093/bioinformatics/btv566. 26428292 PMC4708105

[evae217-B68] Olivier Brun L, Maxwell Suckling D. Field selection for endosulfan resistance in coffee berry borer (Coleoptera: Scolytidae) in New Caledonia. J Econ Entomol. 1992:85(2):325–334. 10.1093/jee/85.2.325.

[evae217-B69] Orozco-Arias S, Sierra P, Durbin R, González J. MCHelper automatically curates transposable element libraries across species. bioRxiv 562682. 10.1101/2023.10.17.562682, 20 October 2023, preprint: not peer reviewed. PMC11694758 39653419

[evae217-B70] Paysan-Lafosse T, Blum M, Chuguransky S, Grego T, Pinto BL, Salazar GA, Bileschi ML, Bork P, Bridge A, Colwell L, et al InterPro in 2022. Nucleic Acids Res. 2023:51(D1):D418–D427. 10.1093/nar/gkac993. 36350672 PMC9825450

[evae217-B71] Petersen M, Armisén D, Gibbs RA, Hering L, Khila A, Mayer G, Richards S, Niehuis O, Misof B. Diversity and evolution of the transposable element repertoire in arthropods with particular reference to insects. BMC Evol Biol. 2019:19(1):1–15. 10.1186/s12862-018-1324-9. 30626321 PMC6327564

[evae217-B72] Puillandre N, Dupas S, Dangles O, Zeddam J-L, Capdevielle-Dulac C, Barbin K, Torres-Leguizamon M, Silvain J-F. Genetic bottleneck in invasive species: the potato tuber moth adds to the list. Biol Invasions. 2008:10(3):319–333. 10.1007/s10530-007-9132-y.

[evae217-B73] Qiu S, Yong L, Wilson A, Croft DP, Graham C, Charlesworth D. Partial sex linkage and linkage disequilibrium on the guppy sex chromosome. Mol Ecol. 2022:31(21):5524–5537. 10.1111/mec.16674. 36005298 PMC9826361

[evae217-B74] Quinlan AR, Hall IM. BEDTools: a flexible suite of utilities for comparing genomic features. Bioinformatics. 2010:26(6):841–842. 10.1093/bioinformatics/btq033. 20110278 PMC2832824

[evae217-B75] R Core Team . A language and environment for statistical computing. Vienna (Austria): R Foundation for Statistical Computing. V4.0.2; 2020. https://www.R-project.org/.

[evae217-B76] Reid JC . Distribution of the coffee berry borer (*Hypothenemus hampei*) within Jamaica, following its discovery in 1978. Trop Pest Manag. 1983:29(3):224–230. 10.1080/09670878309370806.

[evae217-B77] Sambrook J, Russell D. 2001. Molecular cloning: a laboratory manual, Sambrook J, editor. 3rd ed. Vol 3. AbeBooks, 9780879695774, https://www.abebooks.com/9780879695774/Molecular-Cloning-Laboratory-Manual-Third-0879695773/plp (Accessed November 28, 2023).

[evae217-B78] Schrader L, Schmitz J. The impact of transposable elements in adaptive evolution. Mol Ecol. 2019:28:1537–1549. 10.1111/mec.14794. 30003608

[evae217-B79] Schrieber K, Lachmuth S. The genetic paradox of invasions revisited: the potential role of inbreeding × environment interactions in invasion success. Biol Rev Camb Philos Soc. 2017:92(2):939–952. 10.1111/brv.12263. 27009691

[evae217-B80] Simão FA, Waterhouse RM, Ioannidis P, Kriventseva EV, Zdobnov EM. BUSCO: assessing genome assembly and annotation completeness with single-copy orthologs. Bioinformatics. 2015:31(19):3210–3212. 10.1093/bioinformatics/btv351. 26059717

[evae217-B81] Stapley J, Santure AW, Dennis SR. Transposable elements as agents of rapid adaptation may explain the genetic paradox of invasive species. Mol Ecol. 2015:24(9):2241–2252. 10.1111/mec.13089. 25611725

[evae217-B82] Tajima F . Statistical method for testing the neutral mutation hypothesis by DNA polymorphism. Genetics. 1989a:123(3):585–595. 10.1093/genetics/123.3.585. 2513255 PMC1203831

[evae217-B83] Tajima F . The effect of change in population size on DNA polymorphism. Genetics. 1989b:123(3):597–601. 10.1093/genetics/123.3.597. 2599369 PMC1203832

[evae217-B84] Tsutsui ND, Suarez AV, Holway DA, Case TJ. Reduced genetic variation and the success of an invasive species. Proc Natl Acad Sci. 2000:97(11):5948–5953. 10.1073/pnas.100110397. 10811892 PMC18539

[evae217-B85] Vega FE, Brown SM, Chen H, Shen E, Nair MB, Ceja-Navarro JA, Brodie EL, Infante F, Dowd PF, Pain A. Draft genome of the most devastating insect pest of coffee worldwide: the coffee berry borer, *Hypothenemus hampei*. Sci Rep. 2015:5(1):12525. 10.1038/srep12525. 26228545 PMC4521149

[evae217-B86] Vega FE, Kramer M, Jaramillo J. Increasing coffee berry borer (Coleoptera: Curculionidae: Scolytinae) female density in artificial diet decreases fecundity. J Econ Entomol. 2011:104(1):87–93. 10.1603/ec10353. 21404844

[evae217-B87] Walker BJ, Abeel T, Shea T, Priest M, Abouelliel A, Sakthikumar S, Cuomo CA, Zeng Q, Wortman J, Young SK, et al Pilon: an integrated tool for comprehensive microbial variant detection and genome assembly improvement. PLoS One. 2014:9(11):e112963. 10.1371/journal.pone.0112963. 25409509 PMC4237348

[evae217-B88] Witter-Newell D . Development of resistance to insecticides by coffee berry borer, *Hypothenemus hampei* Ferrari (Coleoptera: Scolytidae), in Jamaica and the ecological impact of selected insecticides on the terrestrial fauna of two coffee plantations. 2008. https://hdl.handle.net/2139/2591 (Accessed November 19, 2023).

[evae217-B89] Woronik A, Tunström K, Perry MW, Neethiraj R, Stefanescu C, Celorio-Mancera MP, Brattström O, Hill J, Lehmann P, Käkelä R, et al A transposable element insertion is associated with an alternative life history strategy. Nat Commun. 2019:10(1):5757. 10.1038/s41467-019-13596-2. 31848330 PMC6917731

[evae217-B90] Xia B, Zhang W, Zhao G, Zhang X, Bai J, Brosh R, Wudzinska A, Huang E, Ashe H, Ellis G, et al On the genetic basis of tail-loss evolution in humans and apes. Nature. 2024:626:1042–1048. 10.1038/s41586-024-07095-8. 38418917 PMC10901737

