# Supplementary material for: Genetic Variation in Jamaican Populations of the Coffee Berry Borer, Hypothenemus hampei
Source: Genome Biol Evol. 2024 Nov 1;16(11):evae217. doi: 10.1093/gbe/evae217 (PMC11529894; doi:10.1093/gbe/evae217)
Supplement: evae217_Supplementary_Data [file evae217_supplementary_data.pdf]

Supplementary Tables for:

Genetic variation in Jamaican populations of the coffee berry borer, *Hypothenemus hampei*

Authors:

Errbii, Mohammed[1*]; Myrie, Ameka[2*]; Robinson, Dwight[3]; Schultner, Eva[2]; Schrader, Lukas[1$]; Oettler, Jan[2$]

[*]shared first authors

[$]shared last authors

Affiliation:

[1]Institute for Evolution and Biodiversity, University Münster, 48149 Germany

[2]Zoologie/Evolutionsbiologie, Universität Regensburg, 93053 Germany

[3] Department of Life Sciences, The University of the West Indies, Mona, Kingston, Jamaica

Correspondence:

errbiimohammed@gmail.com; joettler@gmail.com

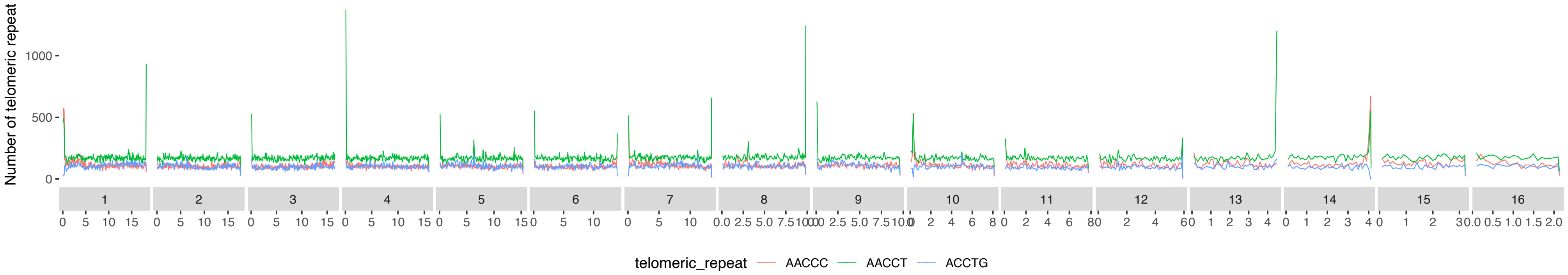

Figure S1. Distribution of known Coleopteran telomeric sequence motifs across the 16 largest scaffolds of Hham4.1. Scaffolds 1, 6, and 7 are enriched in telomeric repeats at both ends, suggesting they may correspond to three of the 14 chromosomes of *Hypothenemus hampei*.

Table S1. Quality metrics of the sequenced pools of the Coffee Berry Borer from different localities in Jamaica.

Collected from:	Abbr./ Code	Pool Size	Insert Size	Median coverage	Mean coverage	% Aligned	Locality
Baron Hall	BH	40	368	61.0X	73.6X	97.20%	Highland
Kew Park	KP	21	342	57.0X	68.9X	96.20%	Lowland
Mocho	MO	33	344	46.0X	61.5X	80.10%	Highland
Rosehill	RH	40	356	65.0X	80.1X	94.40%	Blue Mountains

Table S2. QUAST based statistics calculated for the Hham4.1 genome assembly.

Statistics without reference	Hham4.1
# contigs	114
# contigs (>= 0 bp)	114
# contigs (>= 1000 bp)	114
# contigs (>= 5000 bp)	100
# contigs (>= 10000 bp)	82
# contigs (>= 25000 bp)	65
# contigs (>= 50000 bp)	40
Largest contig	18164326
Total length	172680286
Total length (>= 0 bp)	172680286
Total length (>= 1000 bp)	172680286
Total length (>= 5000 bp)	172634394
Total length (>= 10000 bp)	172500623
Total length (>= 25000 bp)	172202942
Total length (>= 50000 bp)	171283525
N50	13972216
N75	9626410
L50	6
L75	9
GC (%)	32.58
Mismatches	
# N's	0
# N's per 100 kbp	0

Table S3. Comparison of Hham4.1 and previously published CBB genomes.

**Genome Assembly Statistics**	**Published Genomes**			Hham4.1
	Vega et al., 2015	Hernandez-Hernandez et al., 2017	Navarro- Escalante et al. 2021	
Size	163 Mb	197.3/218.3 Mb	162.6 Mb	172.7 Mb
Longest Scaffold	0.44 Mb	-	4.9 Mb	18.2 Mb
GC Content	32.46%	36/38 %	32.32%	32.60%
N50 Contigs	0.011 Mb	0.007/0.028 Mb	0.036 Mb	14 Mb
TE Content	2.7%	8.29%	-	28.89%
Sequencing Technology	Short reads (Illumina Hiseq2000)	Short reads (shotgun seq/ tag- based next gen. Illumina seq)	Short reads (454 and Illumina HiSeq)	Long reads (MinION sequencing)
Origin	Colombian population kept in the lab for ~10 years	Colombian population kept in the lab for ~9 years	Colombian population kept in the lab for more than10 years	Jamaican field population

Table S4. Relative genome proportions of the main repeat families identified using *dnaPipeTE* with raw short reads of Colombian *H. hampei*. The samples were obtained from NCBI (accession numbers SRR11579638 and SRR11579639 (Navarro-Escalante et al. 2021). Note that using this reference-free method revealed a repeat content of approximately 30% in the Colombian strain, which is substantially higher than previously reported values.

Main repeat families	SRR11579638 (1.5Kb-insert Illumina MP 100bp from pooled-female DNA)	SRR11579639 (300bp-insert Illumina PE 100bp from pooled-male DNA)	Average
LTR	1.05	2.30	1.67
LINE	4.38	3.89	4.13
DNA	7.75	3.77	5.76
Helitron	2.39	3.37	2.88
rRNA	0.30	0.21	0.26
Low_Complexity	0.05	0.05	0.05
Satellite	0.002	0.001	0.002
Simple_repeat	0.31	2.71	1.51
others	5.46	8.18	6.82
na	4.67	7.45	6.06
Single copy DNA	73.64	68.07	70.86

Table S5. Mean variation at synonymous and non-synonymous sites in populations of the Coffee Berry Borer from different localities in Jamaica.

	variation at synonymous sites		variation at nonsynonymous sites	
**Population**	**mean**	**sd**	**mean**	**sd**
BH	2.15E-05	2.32E-04	1.48E-05	5.98E-05
KP	2.25E-05	1.64E-04	1.35E-05	4.43E-05
MO	2.55E-05	1.85E-04	2.08E-05	9.45E-05
RH	1.69E-05	1.01E-04	1.41E-05	5.14E-05

Table S6. Mean genetic divergence between Coffee Berry Borer populations from different localities in Jamaica.

**pair**	**Mean *d*xy**	**sd**
BH-vs-RH	1.19E-05	7.29E-05
BH-vs-KP	1.07E-05	7.44E-05
KP-vs-RH	1.19E-05	7.73E-05
MO-vs-KP	1.03E-04	1.43E-04
MO-vs-BH	1.03E-04	1.40E-04
MO-vs-RH	1.04E-04	1.43E-04

References

Hernandez-Hernandez EM et al. 2017. Genome-wide analysis of transposable elements in the coffee berry borer *Hypothenemus hampei* (Coleoptera: Curculionidae): description of novel families. Mol Genet Genomics. 292:565–583. doi: 10.1007/s00438-017-1291-7.

Navarro-Escalante L et al. 2021. A coffee berry borer (*Hypothenemus hampei*) genome assembly reveals a reduced chemosensory receptor gene repertoire and male-specific genome sequences. Sci Rep. 11:4900. doi: 10.1038/s41598-021-84068-1.

Vega FE et al. 2015. Draft genome of the most devastating insect pest of coffee worldwide: the coffee berry borer, *Hypothenemus hampei*. Sci Rep. 5:12525. doi: 10.1038/srep12525.
